# Atopy in Kashmir-validation from a case control study with respect to IgE and Interleukin genes

**DOI:** 10.1186/s13223-021-00623-5

**Published:** 2021-11-23

**Authors:** Taha Ashraf Qurashi, Aaliya Shah, Gulzar Ahmad Bhat, Mosin Saleem Khan, Roohi Rasool, Syed Mudassar

**Affiliations:** 1 Department of Clinical Biochemistry, Sher-I-Kashmir Institute of Medical Sciences, Srinagar, 190011 India; 2 Department of Biochemistry, SKIMS Medical College, Srinagar, 190006 India; 3 Department of Immunology and Molecular Medicine, SKIMS, Srinagar, 190011 India

**Keywords:** IgE, Interleukin gene variants, Atopy, Case control study, Kashmir

## Abstract

**Objectives:**

Increased levels of serum Immunoglobulin-E (IgE) and different genetic variants of cytokines are common biochemical manifestation in Allergy. The current study was aimed to study the association of IgE and different variants of Interleukin-4 (*IL-4)*, and Interleukin-13 (*IL-13*) genes with different kind of allergies.

**Methods:**

A pre-tested questionnaire was used to collect all the dietary, life style and clinical details by a trained staff. A blood sample of 2 ml each was collected in coagulated and anti-coagulated vials. DNA and serum samples were extracted and stored until further use. Serum IgE were estimated by ELISA while as the genotypic analysis was done by PCR–RFLP methods.

**Results:**

**S**tatistically a significant difference of serum IgE levels were observed among cases and controls (*P* < 0.05). The observed significant difference of serum IgE levels were retained among subjects who also harboured variant genotypes of *IL-4* and *IL-13* genes (*P* < 0.05). Additionally, the above genetic variants significantly modified the risk of allergy when stratification was done based on various clinical characteristics.

**Conclusion:**

Our study suggests that increased IgE levels and in association with variant forms of *IL-4* and *IL-13* genes are significantly associated with different types of allergies in study population.

## Introduction

Allergic disorders are considered an epidemic in the developed world with rising incidence rate in the developing countries as well [1–3]. These disorders though multi-etiological but none has been established as a causative factor individually until now. The increased prevalence associated with large differences between urban and rural regions, hints at environmental factors but genetics play a key role independently as well as in combination by modifying environmental and life style exposures.

The condition that result due to exposure of body to environmental substances is characterized by raised serum Immunoglobulin-E (IgE) levels- a feature of allergic diseases. It results due to immune reaction to an antigen called an allergen, which is normally harmless [4, 5]. Allergen-specific IgE- antibodies cause most allergic reactions. A number of atopy susceptibility interleukin genes have been identified which regulate the production of IgE [6, 7]. Genome wide Association studies have identified strong linkage of a region on chromosome 5q31–33 with atopic disorders in different populations [8–11]. Different interleukins (IL) like *IL-4* and *IL-13* genes been reported to regulate IgE synthesis and hence potentially involved in manifestation of allergic disorders [12–14]. The genetic polymorphisms in *IL-4* and *Il-13* genes are the most common genetic alteration in patients having atopic disorders. A number of studies regarding the different polymorphic variants of inflammatory genes and different allergic phenotypes are available [12, 14, 15]. Discovery of these genetic alterations has created the opportunity to develop novel clinical strategies for the management and treatment of these patients.

A number of studies regarding the different polymorphic variants of inflammatory markers and allergic phenotypes are available [12, 14, 15]. The hyperactive immune response in the form of increased levels of IgE and altered IL genetic variants as a common biochemical manifestation among allergic patients has been reported previously [16–20]. Considering, the above findings and possibility of higher incidences of allergies due tosuitable environmental settings for allergies in the form of fluffy seeds from poplar trees and blooms in Kashmir, we intend to identify the patients having different types of allergic disorders, elucidate their serum IgE levels, and find out any possible correlation with certain gene variants of *Il-4* and *IL-13*.

## Materials and methods

### Patients and controls

A total of 528 subjects presented with different allergic symptoms attending Allergy Clinic, Sher-i-Kashmir Institute of Medical Sciences (SKIMS), Department of Dermatology, SKIMS Medical College and Department of Dermatology, Sheri Maharaja Hari Sing (SMHS) Hospital Srinagar were included in this study. A subject is diagnosed as “Atopic” if they have presence of any/all of the phenotypes like; allergic rhinitis (AR), atopic dermatitis (AD), and bronchial asthma (BA) called as allergic triad. Persons with Atopy also have a tendency to have allergic conjunctivitis, food allergy and occupational allergy apart from the above phenotypes. Blood samples were collected from all 528 cases. Blood samples were also obtained from 610 no-atopic subjects from the Out Patients Departments of above Departments. A written pre-informed consent was obtained from all cases and controls. The study was approved by the Institutional Ethical Clearance Committee of SKIMS, Srinagar vide order No. SIMS 1 131/IEC-SKIMS/2016–133.

### Case control selection and data collection

The subject selection was based on the following.

#### Inclusion criteria

All individuals with AD, AR, and BA were included in the study as cases. Patients with allergic conjunctivitis, food allergy, and occupational allergy were also included in the study as cases, in addition to AD, AR, and BA. Those who did not have any of the aforementioned allergic problems served as control subjects.

#### Exclusion criteria

Individuals with any genetic disorder, malignancy, chronic obstructive pulmonary disease and acute chronic obstructive pulmonary disease were excluded from the study. Individuals on beta-blockers and/or with any sort of autoimmune disorder are not eligible for immunotherapy, so they were also excluded from the study.

#### Data collection

Detailed information on age, sex, place of residence, ethnicity, religion, education, dietary data and other potential confounding factors of interest was collected using a questionnaire pre-designed for the study population. Detailed information on life-long history of use of several tobacco products was obtained. Information on family history of any allergic disorder was obtained from all the participants. To assess the socio-economic status (SES) of the subjects, information on potential parameters of SES was obtained including education level (highest level attained), monthly income (INR), house type and ownership of several household appliances. Similarly, the information regarding, second hand smoking was also acquired from all the recruited subjects.

#### Specimen

After obtaining verbal and written consent from all cases and controls, at least 4 ml of blood sample was collected from both cases and controls in EDTA and red top vials. Serum and whole blood sample containing vials were stored at − 20 °C until further use. Serum samples were used to measure the IL-4 and IL-13 levels by Enzyme linked Immunosorbent assay (ELISA). Whole blood samples were used for DNA extraction followed by polymorphic analysis of *IL-4*, *IL-13* and *STAT6* genes.

#### Skin prick test (SPT)

European Academy of Allergy and Clinical Immunology (EAACI) recommendations were used to perform SPT. Saline was used as negative control and histamine 10 mg/ml was used as positive control. Antihistamines were discontinued before skin testing according to published guidelines. Skin tests were considered positive if at least one allergen elicited a wheal reaction of more than 3 mm of diameter after subtraction of the negative control. Patients were considered atopic if at least they had one positive skin test result.

### Estimation of Biochemical and Hematological markers

Serum samples were used for estimation of IgE and Vitamin D levels on Access 2 Immunoassay System (Beckman Coulter, Inc., USA) using CLIA technique. Whole blood samples were used for estimation of eosinophil count on Sysmex XP-300™ Automated Hematology Analyzer (Sysmex America, Inc., USA) and Erythrocyte sedimentation rate (ESR) using Westergren’ method. IgE level of > 180 IU/ml was taken as “Elevated”. In case of Vitamin D levels, < 20 ng/ml was considered as “Deficient”, 20–30 ng/ml as “Insufficient”, 31–100 ng/ml as “Sufficient” and > 100 ng/ml as “Upper safety limit”. Relative eosinophil count > 6% of peripheral blood was considered as “Elevated”. ESR of > 15 mm/hr in males; > 20 mm/hr in females; > 10 mm/hr in children was taken as “Elevated”.

### Pulmonary function test

A spirometry test was performed to access the pulmonary function of each enrolled patient. Forced expiratory volume in 1^ST^ second (FEV1) is a very good and direct indicator of lung function. FEV1 of less than 80% was considered as “Abnormal”. Post Broncho dilatory Response (PBD) is change in FEV1 after 15–20 min of use of Bronchodilator (Salbutamol; 200 µg). PBD of “ > 200 ml and > 12%” is significant for a Reversible Lung disease like Asthma. If PBD is “ < 200 ml and < 12%” the reversibility is not significant as we see in Chronic Pulmonary Obstructive Disease (COPD) and Asthma COPD Overlap Syndrome (ACOPD). Forced vital capacity (FVC) is an indicator of Restrictive lung disease hence not included in our analysis.

### ELISA principle and method

The ELISA was done as described previously [21]. Briefly, a capture antibody highly specific for IL-4 and IL-13 were coated to the wells of the microtiter strip plate. Binding of IL-4 and IL-13 in samples and known standards to the capture antibodies was completed and any excess unbound analyte was removed. During the next incubation period the binding of the biotinylated anti- IL-4 and IL-13 secondary antibody to the analyte occurs. Any excess unbound secondary antibody was then removed. The HRP conjugate solution was then added to every well including the zero wells. Following incubation excess conjugate was removed by careful washing. A chromogen substrate was added to the wells resulting in the progressive development of a blue coloured complex with the conjugate. The colour development was then stopped by the addition of acid turning the resultant final product yellow. The intensity of the produced coloured complex is directly proportional to the concentration of IL-4 and IL-13 present in the samples and standards. The absorbance of the colour complex was then measured and the generated OD values for each standard are plotted against expected concentration forming a standard curve. This standard curve can then be used to accurately determine the concentration of IL-4 and IL-13 in as many samples tested. The primary wavelengths for IL-4 and IL-13 measurement were 450 nm and 630 nm, respectively.

### Sensitivity

The sensitivity or minimum detectable dose of IL-4/IL-13 using diaclone IL-4/IL-13 ELISA kit was found to be 0.3 pg/ml and 1.5 pg/ml respectively. This was determined by adding 3 standard deviations to the mean OD obtained when the zero standard was assayed 3–5 times. However, we had considered 0.7 pg/ml as the minimum value below which the interleukin levels were treated as undetectable in order to be in line with most of the currently used moderately sensitive ELISA kits.

### Genetic analysis

Besides environmental and lifestyle factors, genetics factors also play an important role in the development of allergic disorders. Genetic variants of these interleukin genes have previously been associated with different allergic phenotypes [22] and a detailed account of different gene variants of *IL-4* and *IL-13* have been done as discussed elsewhere [23]. For identification of the studied genotypes, PCR–RFLP analysis was performed [24], the details of which are provided in Table 1.

**Table 1:** Frequency distribution of selected demographic and risk factors in atopy cases and controls of the study

Characteristics	Cases	Controls	*P*_*value*_
	(n = 528)	(n = 610)	
Age years			
< 40	414 (78.4)	459 (75.0)	
> 40	114 (21.6)	151 (25.0)	> 0.05
Mean age	29.7 ± 13.9	29.45 ± 14.2	
Gender			
Male	210 (39.8)	255 (41.8)	
Female	318 (60.2)	355 (58.2)	> 0.05
Dwelling			
Urban	240 (45.5)	281 (46.1)	
Rural	288 (54.5)	329 (44.9)	> 0.05
Smoking status			
Smokers	63 (12.0)	38 (6.2)	> 0.05
Non-smokers	165 (31.2)	345 (56.5)	
Passive smokers	300 (56.8)	227 (37.3)	
IgE levels			
Normal (< 180u/l)	03 (0.6)	487 (79.8)	** < 0.05**
Elevated (≥ 180u/l)	525 (99.4)	123 (20.2)	
Eosinophil count			
Normal (1.0–6.0%)	297 (56.3)	519 (85.1)	
Elevated (> 6.0%)	231 (43.7)	91 (14.9)	> 0.05
Vitamin D levels (ng/ml)			
Deficient (< 20)	345 (65.3)	503 (82.4)	> 0.05
Insufficient (20–30)	111 (21.0)	49 (8.0)	
Sufficient (31–100)	72 (13.7)	58 (9.6)	
Upper safety limit (> 100)	00 (0.0)	00 (0.0)	
ESR			
Normal (< 15 mm/hr in males and < 20 in females)	414 (78.4)		
Elevated (≥ 15 mm/hr in males and ≥ 20 in females)	114 (21.6)		
PFT			
Normal	324 (61.4)		
Abnormal	204 (38.6)		
SPT			
Positive to HDM	72 (13.6)		
Positive to pollens	39 (7.3)		
Positive to HDM/pollens	114 (21.6)		
Positive to HDM/pollens/fungi /AE	66 (12.5)		
Positive to HDM/pollens/few foods/AE	225 (42.6)		
Negative	12 (2.2)		
Socio economic status			
Good	150 (28.4)		
Average	336 (63.6)		
Poor	42 (8.0)		
Seasonal/year round			
Year round	387 (73.3)		
Seasonal	141 (26.7)		
Peak time			
Morning	255 (48.2)		
Evening	249 (47.1)		
Both	24 (4.5)		
Family history			
Yes	393 (74.5)		
No	135 (25.5)		
Triggers			
Dust	174 (32.9)		
Dust/irritant	210 (39.7)		
Temperature	12 (2.3)		
Irritants	132 (25.0)		
Co-morbidity			
Diabetes	24 (4.5)		
Hypertension	27 (5.1)		
Diabetes/hypertension/obesity	16 (2.7)		
Hypothyroidism	51 (9.6)		
PCOD	21 (3.9)		
Nil	389 (73.6)		
Collateral diseases			
Drug allergy	60 (11.5)		
Migraine	120 (22.7)		
Food allergy	90 (17.0)		
Migraine/food allergy	09 (1.7)		
Migraine/drug allergy	06 (1.1)		
Nil	243 (46.0)		
Diagnosis			
AR/BA	210 (39.7)		
AR/AD	57 (10.8)		
AR/CU	36 (6.8)		
AR/BA/AD	120 (22.7)		
AR/BA/CU	75 (14.2)		
AR/BA/conjunctivitis	12 (2.3)		
BA/CU	18 (3.4)		

*IgE* Immunoglobulin E, *GAD* General anxiety disorder, *MDD* Major depressive disorder, *ESR* Eosinophil sedimentation rate, *PFT* Pulmonary function test, *HDM* House dust mite, *AE* Animal epithelia, *AR* Atopic rhinitis, *BA* Bronchial asthma, *AD* topic dermatitis, *CU* Chronic urticarial

### Statistical analysis

Different categorical variables were set for calculating number and percentages for different genotypes of studied genes. Test for Hardy–Weinberg Equilibrium (HWE) were conducted by comparing observed and expected genotype distributions by the χ2 goodness of fit. Statistical significance for departure of a genotype frequency from its expected frequency under the HWE model was set at *P* ≤ 0.05. Conditional logistic regression models were used to calculate odds ratios (ORs) and corresponding 95% confidence intervals (95%CIs) to assess the association of the genotypes with allergies. All statistical analysis was done using STATA software, version 14 (STATA Corp., College Station, TX, USA). Two sided *P* values < 0.05 were considered as statistically significant.

## Results

### Characteristics of study subjects

In this study 60.2% (n = 318) of the cases were females and 39.8% (n = 210) were males with a male:female ratio of 0.7:1. On the basis of age, the patients were grouped into two categories, less than 40 (< 40) and greater or equal to 40 (≥ 40) years of age. The number of cases in the age group of < 40 (n = 414; 78.4%) exceeded as compared to ≥ 40 years (n = 114; 21.6%) with an overall mean age of 29.7 ± 13.9 years. Based on the smoking status, 63 (12%) patients were smokers who were inclusively males, 165 (31.2%) were non-smokers and 300 (56.8%) were passive smokers. There were 525 (99.4%) patient with elevated (≥ 180u/l) IgE levels while as only 3 (0.6%) patients had normal (< 180u/l) IgE levels. Other study variables of the study subjects with atopy are shown in Table 1*.*

Among controls, 58.2% (n = 355) of the subjects were females and 41.8% (n = 255) were females. The number of control subjects in the age group of < 40 (n = 459; 75.0%) exceeded than ≥ 40 years (n = 151; 25.0%). The mean age of the control subjects was 29.45 ± 14.2 years. Based on the smoking status, 38 (6.2%) controls were smokers who were inclusively males, 345 (56.5%) were non-smokers and 227 (37.3%) were passive smokers. There were 123 (20.2%) controls with elevated IgE levels while as 487 (79.8%) had normal IgE levels. Other clinicopathological and clinico-epidemiological characteristics of controls are shown in Table 1*.*

There were no significant differences among cases and controls in terms of mean age, gender distribution and smoking, although, more non-smokers were present in controls than in cases (56.5% vs 31.2%). Similarly, substantially higher number of case (99.4%) showed increased serum IgE levels than controls (20.2%).

### Serum cytokine levels (IL-4 & IL-13) in cases and controls

In the present study we analyzed the serum IL-4 and IL-13 levels in cases and controls in order to evaluate their association with the risk of Atopy in Kashmiri population.

### Serum IL-4 levels and their association with demographic and clinicopathological characteristics of cases and controls

The frequency of detectable IL-4 levels was found to be higher for cases (408 out of 528 i.e. 77.3%) than controls (117 out of 610 i.e. 19.1%), a difference which is statistically significant (*P* < *0.01*)*.* The median IL-4 levels in cases and controls was found to be 2.57 (0.0–23.35) and 0.08 (0.0–4.37) respectively (Table 2)*.*

**Table 2:** The number of subjects with detectable serum *IL-4* and *IL-13* levels along with its median and range in patient and control group

Cytokines (pg/mL)	Cases		Controls		*P* value
	N (%)	Median (min–max)	n (%)	Median (min–max)	
*IL-4*	408/528 (77.3)	2.57 (0.0–23.35)	117/610 (19.1)	0.08 (0.0–4.37)	< 0.01
*IL-13*	528/528 (100)	13.79 (5.98–42.47)	395/610 (64.7)	2.32 (0.0–6.51)	< 0.01

On further stratification, association between IL-4 levels and clinico-pathological variables was analyzed between cases and controls. It was observed that there was a statistically significant difference in the frequency of detectable and undetectable IL-4 levels between cases and controls with respect to all the subgroups of IgE levels, age, gender, dwelling, smoking status, eosinophil count and vitamin D levels (*P* < 0.01) (Table 3)*.*

**Table 3:** Correlation of various clinico-pathological characteristics of cases and controls with serum *IL-4* levels

Variables	Cases			**Controls**			**OR (95%CI)**	P Value
	Number (n)	Un (%)	**Dn (%)**	**Number (n)**	**Un (%)**	**Dn (%)**		
	n = 528	120 (22.7)	408 (77.3)	n = 610	493 (80.9)	117 (19.1)	14.3 (10.7–19.0)	** < 0.0001**
Age								
< 40	414 (78.4)	102	312	459 (75.0)	373	86	13.2 (9.5–18.3)	** < 0.0001**
> 40	114 (21.6)	18	96	151 (25.0)	120	31	20.6 (10.8–39.1)	** < 0.0001**
Gender								
Male	210 (39.8)	36	174	255 (41.8)	205	50	19.8 (13.3–31.8)	** < 0.0001**
Female	318 (60.2)	84	234	355 (58.2)	288	67	11.9 (8.3–17.2)	** < 0.0001**
Dwelling								
Urban	240 (45.5)	48	192	281 (46.1)	221	60	14.7 (9.6–22.5)	** < 0.0001**
Rural	288 (54.5)	72	216	329 (44.9)	272	57	14.3 (9.6–21.1)	** < 0.0001**
Smoking status								
Smokers	63 (12.0)	12	151	38 (6.2)	32	06	67.1 (23.4–92.0)	** < 0.0001**
Non-smokers	165 (31.2)	42	123	345 (56.5)	187	40	1.36 (8.3–22.3)	** < 0.0001**
Passive smokers	300 (56.8)	66	234	227 (37.3)	274	71	13.6 (9.3–19.9)	** < 0.0001**
IgE levels								
Normal	03 (0.6)	00	03	487 (79.8)	405	82	19.5 (2.1–77.2)	** < 0.0001**
Elevated	525 (99.4)	120	405	123 (20.2)	88	35	8.4 (5.4–13.1)	** < 0.0001**
Eosinophil count								
Normal	297 (56.3)	75	222	519 (85.1)	419	100	12.4 (8.8–17.4)	** < 0.0001**
Elevated	231 (43.7)	45	186	91 (14.9)	74	17	17.9 (9.6–33.4)	** < 0.0001**
Vitamin D levels (ng/ml)								
Deficient (< 20)	345 (65.3)	93	252	503 (82.4)	408	95	11.6 (8.3–16.1)	** < 0.0001**
Insufficient (20–30)	111 (21.0)	15	96	49 (8.0)	40	09	28.4 (11.5–70.3)	** < 0.0001**
Sufficient (31–100)	72 (13.7)	12	60	58 (9.6)	45	13	17.3 (7.2–41.5)	** < 0.0001**
ESR							1.0 (0.6–1.7)	0.7
Normal	414 (78.4)	93	321					
Elevated	114 (21.6)	27	87					
PFT							0.7 (0.4–1.09)	0.1
Normal	324 (61.4)	81	243					
Abnormal	204 (38.6)	39	165					
SPT								0.7
HDM	72 (13.6)	15	57					
Pollens	39 (7.3)	09	30					
HDM/pollens	114 (21.6)	21	93					
HDM/pollens/fungi/AE	66 (12.5)	24	42					
HDM/pollens/few foods/AE	225 (42.6)	48	77					
Negative	12 (2.2)	03	09					
Socio economic status							1.6 (0.98–2.5)	0.06
Good	150 (28.4)	27	123					
Average	336 (63.6)	87	249					
Poor	42 (8.0)	06	36					
Seasonal/year round								
Year round	387 (73.3)	99	288					
Seasonal	141 (26.7)	21	120				0.5 (0.3–0.8)	**0.009**
Peak time							1.3 (0.9–2.0)	0.1
Morning	255 (48.2)	51	204				1.3 (0.5–3.5)	0.3
Evening	249 (47.1)	63	186					
Both	24 (4.5)	06	18					
Family history							1.1 (0.7–1.8)	0.6
Yes	393 (74.5)	87	306					
No	135 (25.5)	33	102					
Triggers								0.2
Dust	174 (32.9)	42	132					
Dust/irritant	210 (39.7)	51	159					
Temperature	12 (2.3)	03	9					
Irritants	132 (25.0)	24	108					
Co-morbidity								
Diabetes	24 (4.5)	03	21					
Hypertension	27 (5.1)	03	24					
Diabetes/hypertension/obesity	16 (2.7)	00	16					
Hypothyroidism	51 (9.6)	15	36					
PCOD	21 (3.9)	03	18					
Nil	389 (73.6)	96	293					**0.02**
Collateral diseases								0.3
Drug allergy	60 (11.5)	12	48					
Migraine	120 (22.7)	30	90					
Food allergy	90 (17.0)	15	75					
Migraine/food allergy	09 (1.7)	06	03					
Migraine/drug allergy	06 (1.1)	00	06					
Nil	243 (46.0)	57	186					
Diagnosis								**0.03**
AR/BA	210 (39.7)	45	165					
AR/AD	57 (10.8)	06	51					
AR/CU	36 (6.8)	09	27					
AR/BA/AD	120 (22.7)	27	93					
AR/BA/CU	75 (14.2)	21	54					
AR/BA/conjunctivitis	12 (2.3)	06	06					
BA/CU	18 (3.4)	06	12					

*IL* Interleukin, *U* undetectable, *D* detectable, *IgE* immunoglobulin E, *GAD* general anxiety disorder, *MDD* major depressive disorder, *ESR* eosinophil sedimentation rate, *LSCS* lower segment caesarian section, *TSH* thyroid stimulating hormone, *PFT* pulmonary function test, *HDM* house dust mite, *AE* animal epithelia, *AR* atopic rhinitis, *BA* bronchial asthma, *AD* topic dermatitis, *CU* chronic urticaria

0.7 pg/ml is the minimum value below which the interleukin levels (IL-4) were treated as undetectable

Frequency of detectable IL-4 levels was more common in cases with year round allergy as compared to patients with seasonal allergy (OR = 0.5, *P* = 0.01), in cases with other co-morbidities (*P* = 0.02) and patients with all the differential diagnosis of Atopy (*P* = 0.03) (Table 3)*.*

When the different studied polymorphisms of *IL-4RA, IL-4, IL-13 and STAT6* genes were compared with IL-4 levels of Atopy patients the statistical significance was noted with *IL-4RA* A148G (OR = 2.0; P = 0.01), *IL-13* A1512C (OR = 0.5; P = 0.05) and *STAT6* G2964A (OR = 0.6; *P* = 0.03) respectively (Table 4)*.*

**Table 4:** Correlation of various genetic variants with serum *IL-4* levels in Atopy patients

Overall genotype	Cases			OR (95%CI)	P value
	Number	U	D		
	(n)	n (%)	n (%)		
	n = 528	120 (22.7)	408 (77.3)		
*IL-4 RA* (A148G)				2.0 (1.1–3.7)	**0.01**
AA	108 (20.4)	15	93		
AG + GG	420 (79.6)	105	315		
*IL-4RA* (A1902G)				1.5 (0.8–2.5)	0.17
AA	102 (19.3)	18	84		
AG + GG	426 (70.7)	102	324		
*IL-4* 70 bp VNTR				0.8 (0.5–1.3)	0.4
2 repeats	93 (17.6)	24	69		
2/3 repeats	435 (72.4)	96	339		
*IL-13* (C1055T)				0.8 (0.5–1.2)	0.4
CC	207 (39.2)	51	156		
CT + TT	321 (60.8)	69	252		
*IL-13* (A1512C)				0.5 (0.3–0.8)	**0.005**
AA	99 (18.7)				
AC + CC	429 (71.3)	33	66		
*STAT-6* (G2964A)				0.6 (0.4–0.95)	**0.03**
GG	156 (29.5)	87	342		
GA + AA	372 (70.5)	75	297		

*IL *Interleukin, *U* undetectable, *D* detectable

0.7 pg/ml is the minimum value below which the interleukin levels (IL-4) were treated as undetectable

1.5 pg/ml is the minimum value below which the interleukin levels (IL-13) were treated as undetectable

### Serum IL-13 levels and their association with demographic and clinicopathological characteristics of cases and controls

Detectable IL-13 levels in Atopy patients and matched controls were analysed in order to evaluate its association with the risk of Atopy in Kashmiri population. All cases had *detectable* IL-13 levels (528 of 528; 100.0%) as compared to controls (395 of 610; 64.7%), a difference which is statistically significant (P < 0.01)*.* The median IL-13 levels in cases and controls were found to be 13.79 (5.98–42.47) and 2.32 (0.0–6.51) respectively (Table 2)*.* There was a significant difference between detectable and undetectable IL-13 levels of cases and controls (P < 0.0001).

On comparing IL-13 with various clinicopathological variables, a statistically significant difference in the frequency of detectable and undetectable IL-13 levels were observed in both the groups with respect to all the subgroups of IgE, age, gender, dwelling, smoking status, levels, eosinophil count and vitamin D levels so consequently there was no overall statistical significance with respect to these clinicopathological characteristics (*P* < 0.0001). However, association between IL-13 levels and demographic/clinicopathological characteristics did not presented any significant association among cases (P > 0.05) (Table 5)*.*

**Table 5:** Correlation of various clinic-pathological characteristics of cases and controls with *IL-13* serum levels

Variables	Cases			Controls			OR (95%CI)	P value
	Number (n)	Un (%)	D	Number	Un (%)	Dn (%)		
			n (%)	(n)				
	n = 528	00 (00.00)	528 (100.0)	n = 610	215 (35.3)	395 (64.7)	–	** < 0.0001**
Age							–	
< 40	414 (78.4)	00	414	459 (75.0)	158	301		** < 0.0001**
> 40	114 (21.6)	00	114	151 (25.0)	57	94		** < 0.0001**
Gender							–	
Male	210 (39.8)	00	310	255 (41.8)	83	172		** < 0.0001**
Female	318 (60.2)	00	318	355 (58.2)	132	223		** < 0.0001**
Dwelling							–	
Urban	240 (45.5)	00	240	281 (46.1)	108	173		** < 0.0001**
Rural	288 (54.5)	00	288	329 (44.9)	107	222		** < 0.0001**
Smoking status							–	
Smokers	63 (12.0)	00	63	38 (6.2)	12	26		** < 0.0001**
Non-smokers	165 (31.2)	00	165	345 (56.5)	80	147		** < 0.0001**
Passive smokers	300 (56.8)	00	300	227 (37.3)	123	222		** < 0.0001**
IgE Levels:							–	
Normal	03 (0.6)	00	3	487 (79.8)	175	312		** < 0.0001**
Elevated	525 (99.4)	00	525	123 (20.2)	40	83		** < 0.0001**
Eosinophil count							–	
Normal	297 (56.3)	00	297	519 (85.1)	183	336		** < 0.0001**
Elevated	231 (43.7)	00	231	91 (14.9)	32	59		** < 0.0001**
Vitamin D levels (ng/ml)							–	
Deficient (< 20)	345 (65.3)	00	345	503 (82.4)	173	330		** < 0.0001**
Insufficient (20–30)	111 (21.0)	00	111	49 (8.0)	18	31		** < 0.0001**
Sufficient (31–100)	72 (13.7)	00	72	58 (9.6)	24	34		** < 0.0001**
ESR							–	0.3
Normal	414 (78.4)	00	414					
Elevated	114 (21.6)	00	114					
PFT							–	0.6
Normal	324 (61.4)	00	324					
Abnormal	204 (38.6)	00	204					
SPT							–	
HDM	72 (13.6)	00	72					
Pollens	39 (7.3)	00	39					
HDM/pollens	114 (21.6)	00	114					
HDM/pollens/fungi /AE	66 (12.5)	00	66					
HDM/pollens/few foods/AE	225 (42.6)	00	225					
Negative	12 (2.2)	00	12					
Socio economic status							–	0.5
Good	150 (28.4)	00	150					0.4
Average	336 (63.6)	00	336					
Poor	42 (8.0)	00	42					
Seasonal/year round							–	0.4
Year round	387 (73.3)	00	387					
Seasonal	141 (26.7)	00	141					
Peak time							–	0.7
Morning	255 (48.2)	00	255					0.1
Evening	249 (47.1)	00	249					
Both	24 (4.5)	00	24					
Family history							–	0.5
Yes	393 (74.5)	00	393					
No	135 (25.5)	00	135					
Triggers							–	–
Dust	174 (32.9)	00	174					
Dust/irritant	210 (39.7)	00	210					
Temperature	12 (2.3)	00	12					
Irritants	132 (25.0)	00	132					
Co-morbidity							–	–
Diabetes	24 (4.5)	00	24					
Hypertension	27 (5.1)	00	27					
Diabetes/hypertension/obesity	16 (2.7)	00	16					
Hypothyroidism	51 (9.6)	00	51					
PCOD	21 (3.9)	00	21					
Nil	389 (73.6)	00	389					
Collateral diseases							–	–
Drug allergy	60 (11.5)	00	60					
Migraine	120 (22.7)	00	120					
Food allergy	90 (17.0)	00	90					
Migraine/food allergy	09 (1.7)	00	9					
Migraine/drug allergy	06 (1.1)	00	6					
Nil	243 (46.0)	0	243					
Diagnosis							–	–
AR/BA	210 (39.7)	00	210					
AR/AD	57 (10.8)	00	57					
AR/CU	36 (6.8)	00	36					
AR/BA/AD	120 (22.7)	00	120					
AR/BA/CU	75 (14.2)	00	75					
AR/BA/conjunctivitis	12 (2.3)	00	12					
BA/CU	18 (3.4)	00	18					

*IL* Interleukin, *U* undetectable, *D* detectable, *IgE* immunoglobulin E, *GAD* general anxiety disorder, *MDD* major depressive disorder, *ESR* eosinophil sedimentation rate, *LSCS* lower segment caesarian section, *TSH* thyroid stimulating hormone, *PFT* pulmonary function test, *HDM* house dust mite, *AE* animal epithelia, *AR* atopic rhinitis, *BA* bronchial asthma, *AD* atopic dermatitis, *CU* chronic urticaria

1.5 pg/ml is the minimum value below which the interleukin levels (IL-13) were treated as undetectable

When the different studied polymorphisms of *IL-4Rα, IL-4, IL-13 and STAT6* genes were compared with IL-13 levels of Atopy patients the statistical significance was not noted with any of the polymorphisms (Table 6)*.*

**Table 6:** Correlation of various genetic variants with *IL-13* levels of cases and controls

Overall genotype/	Cases			OR (95%CI)	P value
IL-4 levels	Number (n)	Un (%)	D n (%)		
	n = 528	00 (00.0)	528 (100.0)		
*IL-4* RA (A148G)				0.2 (0.01–4.1)	0.4
AA	108 (20.4)	00	108		
AG + GG	420 (79.6)	00	420		
*IL-4RA* (A1902G)				0.2 (0.01–3.8)	0.3
AA	102 (19.3)	00	102		
AG + GG	426 (70.7)	00	426		
*IL-4* 70 bp VNTR				0.2 (0.01–3.4)	0.3
2 repeats	93 (17.6)	00	93		
2/3 repeats	435 (72.4)	00	435		
*IL-13* (C1055T)				0.6 (0.04–10.3)	0.6
CC	207 (39.2)	00	207		
CT + TT	321 (60.8)	00	321		
*IL-13* (A1512C)				0.2 (0.01–3.7)	0.3
AA	99 (18.7)	00	99		
AC + CC	429 (71.3)	00	429		
*STAT-6* (G2964A)					
GG	156 (29.5)	00	156	0.4 (0.02–6.7)	0.5
GA + AA	372 (70.5)	00	372		

*IL* Interleukin, *U* undetectable, *D* detectable

0.7 pg/ml is the minimum value below which the interleukin levels (IL-4) were treated as undetectable

1.5 pg/ml is the minimum value below which the interleukin levels (IL-13) were treated as undetectable

**Fig. 1:**
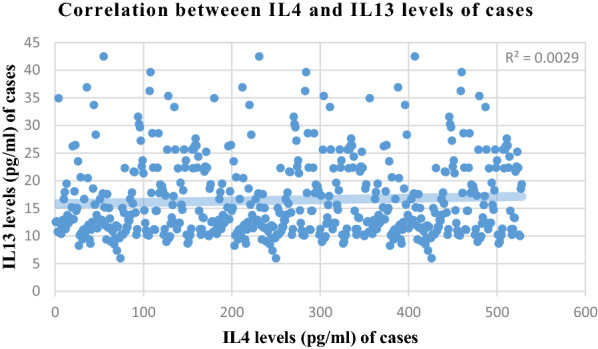
Correlation analysis of various parameters between cases and controls Association is significant with positive but low correlation among all parameters correlated above

**Fig. 2:**
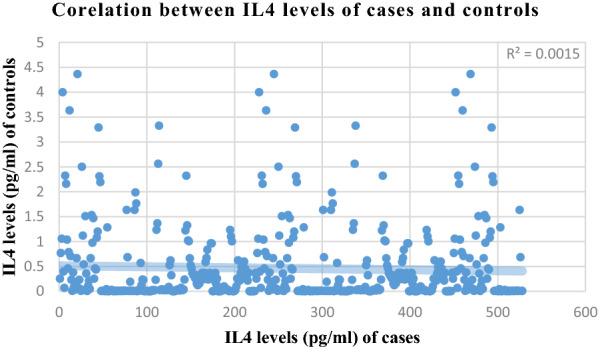
Correlation analysis of various parameters between cases and controls Association is significant with positive but low correlation among all parameters correlated above

**Fig. 3:**
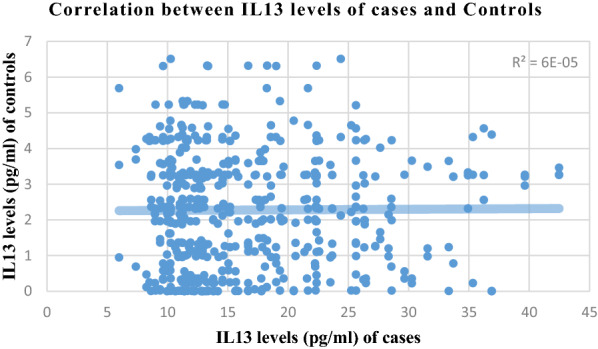
Correlation analysis of various parameters between cases and controls Association is significant with positive but low correlation among all parameters correlated above

**Fig. 4:**
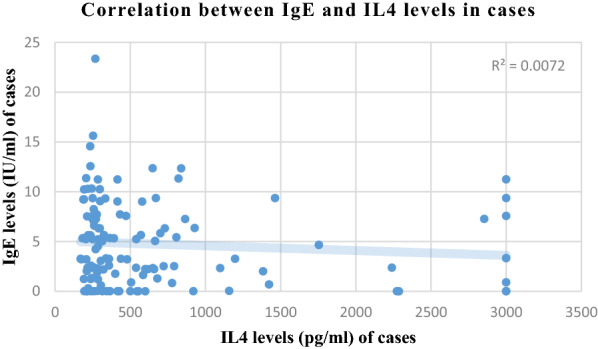
Correlation analysis of various parameters between cases and controls Association is significant with positive but low correlation among all parameters correlated above

Correlation analysis of various parameters between cases and controls is presented in Figs. 1, 2, 3 and 4. Most of the studied parameters showed a positive but significant correlation between cases and controls.

## Discussion

IgE plays an important role in the development of allergic disorders. Current evidence shows that high-affinity IgE in blood stream of allergic individuals derives mainly from the mucous membrane. The production of IgE requires the activation of the adaptive immune system. B cells in the secondary lymphoid organs are stimulated to produce mature antibodies [25]. Moreover, the stimulus upon exposure of allergens in the mucosa might produce a sequential switching to IgE from IgG or IgA [26, 27]. A study from early 1990s demonstrated that house dust mite-specific IgE increased faster in the nasal secretions than in serum after allergen exposure [28]. Similarly allergic rhinites patients on exposure to pollen showed significant changes in IgE repertoires in nasal mucosa [29, 30].

Different cytokines are modulating factors of the immune response and inflammatory reactions and are involved in the pathogenesis of allergies. In this study, we observed a substantial increase in serum IgE levels among atopic subjects as compared to non-atopic controls (*P* < 0.05). In subjects with increased levels of serum IgE levels, we observed higher percentage of variant genotypes of *IL-4* and *IL-13* genes. Additionally, there was an increased serum IL-4 and IL-13 levels among atopic patients as compared to controls.

Our results are completely in agreement with earlier epidemiological studies which reported higher IgE levels associated with bronchial responsiveness, a major component of the asthma phenotype, rhinitis and dermatitis [31–33]. In recent years, a high serum levels of IgE is used to predict the development of asthma, independent of other allergic factors. Therefore, an understanding of the genetic mechanisms regulating total serum IgE levels will be effective to scrutinize the hereditary components of different atopic disorders, complex genetic disorders influenced by the interactions among multiple genes and environmental exposures [34]. In another study, a two-locus segregation analysis revealed evidence of two major gene locus on chromosome 5 (5q31-533) and a residual genetic effect regulating total serum IgE levels in asthma patients [35]. Several studies have found evidence for a recessive gene regulating IgE levels, with different estimates of gene frequencies and mean IgE levels [36].

*IL-13* and *IL-4* genes have been reported to play an important role in allergy [37, 38]. They recruit eosinophils and mast cells, induce B-cells for IgE synthesis [39]. In addition, *IL-4* is also responsible for regulating differentiation of naive T cells into Th2 subtype. In response to allergen IL-4 strongly influence bronchial hyperactivity resulting in airway inflammation, mucus hypersecretion and airway remodelling [20]. Previous studies have shown that SNPs in the *IL-4* promoter may influence the response of mast cells to IgE-mediated signaling [16]. Polymorphism rs2243250 was associated with IgE levels, asthma, rhinitis and dermatitis [40, 41]. The studied polymorphism of *IL-4* has also been associated with different topic disorders like atopic asthma and increased total IgE level [42]. In *IL-13* gene, a functional SNP contributing to amino acid substitution R110Q (rs20541) was described to change the binding strength of *IL-13* to its receptor, causing higher activity of Gln110 variant [17]. Numerous studies showed that this polymorphism correlated significantly with higher total IgE levels among allergic patients [38, 43]. Furthermore, study by Choi et al. describes significant association with an increase in total serum IgE levels in children with atopic asthma in Korean population for rs20541 and rs1805010 polymorphisms [44], which is completely in agreement with our results. In allergic subjects, Th2 phenotype predominates, leading to increased production of serum IL-13 and class-switching in B lymphocytes to synthesize IgE antibodies [45, 46]. Arima et al. have found, using animal model, that *IL-13* with Gln110 variant has higher activity and is present in blood for a longer time [17]. This was confirmed by Chen et al. [47, 48], who performed the experiments on mouse cell line with stable expression of human *IL-13R*, that had the ability to bind human *IL-13* gene. In agreement with the above findings, we observed a significant increase in IgE levels among subjects harboring the variant forms of studied genes.

## Conclusion

The current findings suggest that IgE and interleukin genes play a key role in allergy disease development. Significantly raised serum levels of IgE, as well as both of the study's inflammatory markers were observed among cases as compared to controls. With respect to several subgroups including serum IgE levels, age, gender, residence, smoking status, eosinophil count, and vitamin D levels, there was a significant difference in the frequency of detectable and undetectable IL-4 and IL-13 levels between patients and controls. Only a few genotypes exhibited a significant difference between detectable and undetectable IL-4 serum levels, while IL-13 levels revealed no significant difference.

The strength of the study is that no study has reported the exposure of these genotypes with atopic disorders alone or in combination with these environmental exposures in Kashmiri population. Other major strength is comparatively larger sample size and confounding of the results with the probable allergy risk factors. However, selection or recall bias could be the weak point of this study, although the same hospital setting and only a single interviewer lessen this bias.

## Data Availability

Not applicable.
